# Mutant p53: Multiple Mechanisms Define Biologic Activity in Cancer

**DOI:** 10.3389/fonc.2015.00249

**Published:** 2015-11-11

**Authors:** Michael Paul Kim, Yun Zhang, Guillermina Lozano

**Affiliations:** ^1^Department of Surgical Oncology, The University of Texas MD Anderson Cancer Center, Houston, TX, USA; ^2^Department of Genetics, The University of Texas MD Anderson Cancer Center, Houston, TX, USA

**Keywords:** mutant proteins, p53 mutation, gain of function, stroma, mouse models of cancer, TP53, cancer

## Abstract

The functional importance of *p53* as a tumor suppressor gene is evident through its pervasiveness in cancer biology. The *p53* gene is the most commonly altered gene in human cancer; however, not all genetic alterations are biologically equivalent. The majority of alterations involve *p53* missense mutations that result in the production of mutant *p53* proteins. Such mutant *p53* proteins lack normal *p53* function and may concomitantly gain novel functions, often with deleterious effects. Here, we review characterized mechanisms of mutant *p53* gain of function in various model systems. In addition, we review mutant *p53* addiction as emerging evidence suggests that tumors may depend on sustained mutant *p53* activity for continued growth. We also discuss the role of *p53* in stromal elements and their contribution to tumor initiation and progression. Lastly, current genetic mouse models of mutant *p53* in various organ systems are reviewed and their limitations discussed.

## Mutant *p53*, the Elephant in the Room

Cancer is a complex disease that kills millions of people annually. Alterations in genetic and epigenetic cellular programs derail cellular controls normally responsible for maintaining homeostasis. Sequencing of human cancer genomes has identified a myriad of genomic alterations found in human cancers. Alterations in the *p53* tumor suppressor gene stand out as the most common alteration in many cancers: 96% in ovarian serous carcinoma (1), 54% in invasive breast carcinomas (2), 86% in small cell lung cancer (3), and 75% in pancreas cancer (4), to name a few. Although *p53* activity may be abrogated or lost through multiple mechanisms, the majority of these changes involve *p53* missense mutations that result in single amino acid substitutions and expression of mutant proteins. Common mutations in the *p53* gene, or “hotspots,” are present; for example, approximately 86% of mutations correspond to the DNA-binding sequence of *p53* between codons 125 and 300. The predominance of mutant *p53* protein expression in human cancers over the simple loss of *p53* activity, in turn, suggests an inherent biologic advantage (5–7).

## *p53* Biological Activities in Tumor Suppression

The *p53* gene encodes a transcription factor that contains a potent transcriptional activation domain, a sequence-specific DNA-binding domain, and a tetramerization domain (8). In normal cells, *p53* activity is low, but in response to DNA damage and numerous other stress signals, *p53* levels rise dramatically and result in the activation and transcription of hundreds of genes with important roles in cell cycle arrest, senescence, apoptosis, metabolism, and differentiation (9). The sum of these activities is to ensure that an abnormal cell fails to proliferate. Thus, tumors arise upon depletion of *p53* activity through various mechanisms, including deletion or mutation of the *p53* gene itself, overproduction of the *p53* inhibitors, Mdm2 and Mdm4, and viral inactivation (10–12). Regardless of the mechanism of *p53* loss, the downstream consequences are profound and likely due to the vast, fundamental spectrum of biologic activities in which *p53* normally participates. Moreover, the loss of normal *p53* function is likely coupled with the adoption of new biologic functions exerted by mutant *p53* proteins with additional, deleterious effects.

## Gain-of-Function Activities of Mutant *p53*

Single amino acid changes in the *p53* gene may result in profound changes to its function. In human cancers, missense mutations comprise approximately 75% of all *p53* alterations (7, 13, 14). This is in contrast to many other tumor suppressor genes that undergo deletion through the course of tumor initiation or development, such as *PTEN*, *BRCA1*, and *Rb*. Five arginine residues in the *p53* gene are considered mutational “hotspots”; resultant mutant proteins fail to bind to sequence-specific DNA sites and therefore drastically alter the spectrum of transcriptional activity (15). Such signature mutations in the *p53* gene may arise through environmental exposure to ultraviolet light or chemical carcinogens such as aflatoxins, smoking, and so on (7, 16).

The fact that most *p53* alterations in tumors are missense mutations suggests that cells expressing mutant *p53* have an advantage over cells lacking *p53* (17). Numerous experiments have tested this hypothesis. For example, various tumor-derived human *p53* mutants introduced into *p53-null* H1299 lung adenocarcinoma cells conferred upon tumor cells a selective survival advantage during etoposide or cisplatin treatments (18). In addition, several *p53* mutants when overexpressed in Saos-2 cells, an immortalized tumor cell line that lacks *p53*, yielded tumors in nude mice, while the parental Saos-2 cell line did not (19). Cells expressing the most common *p53* mutants, in contrast to cells lacking *p53*, also show increased metastatic potential and invasiveness (20, 21). Mutant *p53* proteins also render some cell types more resistant to killing by therapeutic drugs such as doxorubicin, etoposide, and cisplatin (22). In Li–Fraumeni syndrome (LFS), individuals with *p53* missense mutations show a higher cancer incidence and an earlier age of tumor onset (9–15 years earlier depending on the study) than individuals with other kinds of mutations (23). These novel activities of mutant *p53* are referred to as gain of function (GOF).

The generation of *p53* knockin alleles in mice provided direct *in vivo* evidence for the GOF activities of mutant *p53*. Knockin mouse models that express mutant *p53*^*R172H*^ and *p53*^*R270H*^ proteins, which mimic hot spot mutations that correspond to amino acids 175 and 273 in human cancers, respectively, develop tumors that exhibit a GOF phenotype *in vivo*, with high metastatic capacity compared to tumors in mice inheriting a *p53*-null allele (24, 25). Additionally, using autochthonous mouse models of pancreatic cancer that incorporate oncogenic K-ras, Morton et al. (26) found no metastatic burden in mice that had undergone genetic deletion of a normal *p53* allele relative to a high (65%) incidence of metastasis in mice expressing a single, mutant *p53* allele (26). However, other groups that have studied identical mouse models of pancreatic cancer have found cells of pancreatic origin in the bloodstream of mice that have undergone monoallelic or biallelic deletion of *p53* in the pancreas, without the presence of mutant *p53* (27–29). These data suggest that mutant *p53* GOF activities may serve to enhance the metastatic potential and/or promote the survival and productivity of metastatic tumor cells at distant sites (26). Taken together, these studies suggest that stable mutant *p53* proteins have additional activities that fuel tumor cell proliferation and metastases that are not yet fully understood.

Interestingly, the characterization of animal models containing mutant *p53* alleles have demonstrated that tumor-specific events were required for the stabilization of mutant *p53* in addition to its simple expression. Numerous tissues derived from mouse models with germline mutant *p53* alleles failed to demonstrate detectable mutant *p53* proteins, and, in some cases, tumors failed to express detectable mutant *p53*. Investigation into this phenomenon concluded that normal tissues failed to stabilize mutant *p53* due to the presence of *Mdm2 and p16^INK4a^*. Upon loss of *Mdm2 or p16^INK4a^*, mutant *p53* is stabilized and mice show decreased survival and increased metastases relative to mice with intact *Mdm2 or p16^INK4a^* alleles (30). A recent analysis of pancreatic cancer specimens demonstrated a strong correlation between *p53* mutation and its stabilization through positive staining by immunohistochemistry for *p53* protein expression. Such data again indicate that in patients with pancreatic cancer, mutant *p53* proteins are expressed, stabilized, and play an important role in tumor development and progression (31). The GOF activity of mutant *p53* therefore depends largely on multiple signals for its stabilization that may vary among normal cells and even among tumor cells.

## Mechanisms of Mutant *p53* GOF

Several mechanisms have now been identified that contribute to mutant *p53* GOF activities. The first such mechanism discovered showed that mutant *p53* proteins abrogate the tumor-suppressive activities of the *p53* family members *p63* and *p73* (24, 25, 32–34). In addition, *TGF*-*β* and *EGFR*/integrin signaling pathways stabilized mutant *p53* (*p53*^*R175H*^ and *p53*^*R273H*^ introduced into *p53*-null H1299 cells) and inhibited the function of *p63*, properties that were essential for the invasive nature of these cells (35, 36). These studies strengthened the evidence that mutant *p53* proteins bind and disrupt *p63* activities. However, *p63* expression is limited to epithelial cells and its inhibition may therefore not explain mutant *p53* GOF in tumors of mesenchymal origin. Moreover, mutant *p53* was found to regulate gene expression independently of *p63* and *p73* in some tumors (37–40).

Using cell lines derived from these same pancreatic cancer models with *Ras* and *p53* mutations, mutant *p53* was found to drive metastasis through induction of platelet-derived growth factor receptor β (*PDGFRβ*). Mutant *p53*-dependent sequestration of *p73* from an *NF-Y* complex permits this transcriptional complex to function at the platelet-derived growth factor β promoter, resulting in expression of *PDGFRβ* and a prometastatic phenotype (41).

**Figure 1:**
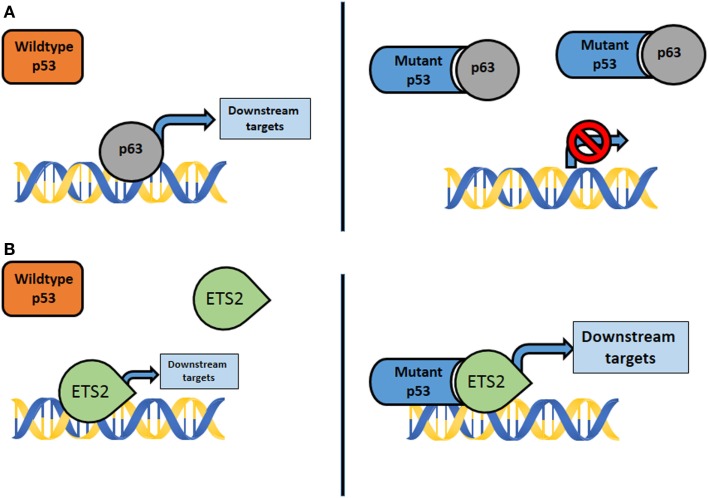
**(A)** Mutant *p53* interacts with transcription factors not normally bound by wildtype *p53*, such as *p63*, *p73*, and *Smad*. The activity of downstream targets is disrupted, resulting in GOF properties. **(B)** Mutant *p53* complexes with transcription factors, such as *Ets2* and *SREBP*, not typically bound by wildtype *p53*. The results are aberrant activation of genes and downstream effectors that promote GOF properties.

Chromatin ImmunoPrecipitation (ChIP)-on-chip experiments and expression arrays using SKBR3 breast cancer cells with the *p53*^*R175H*^ mutation identified mutant *p53* complexes with the vitamin D receptor which augmented expression of survival genes and dampened expression of proapoptotic genes (42). Importantly, in these experiments, an intact transcriptional activation domain was required. Using expression arrays of MDA-MB-468 (*p53*^*R273H*^) breast cancer cells, Freed-Pastor et al. (43) identified increased expression of genes encoding several enzymes of the mevalonate pathway. Mutant *p53* bound *SREBP* proteins and disrupted the acinar architecture of breast epithelial cells when grown as spheroids. In our studies, we compared primary osteosarcomas that had metastasized from *p53*^R172H/+^ mice to *p53*^+/−^ tumors that lacked metastases and identified a unique set of transcriptional changes (39). In this system, mutant *p53* bound the transcription factor *Ets2* and enhanced expression of a phospholipase, *Pla2g16*, which induced migration and invasion in culture (39). Lastly, ChIP-seq experiments using LFS fibroblasts with the *p53*^*R248W*^ mutation identified numerous promoters that contain mutant *p53* (42, 44). More recently, Zhu et al. showed that *p53* mutants, not wild-type (WT) *p53*, bind to and upregulate chromatin regulatory genes, including the methyltransferase *MLL1*, *MLL2*, and acetyltransferase *MOZ*, resulting in genome-wide increases of histone methylation and acetylation. Furthermore, upregulation of *MLL1*, *MLL2*, and *MOZ* was found in human tumors with *p53* mutants, but not in WT *p53* or *p53*-null tumors (45). In summary, these data suggest that multiple pathways contribute to the GOF phenotypes of cells with mutant *p53*. The emerging themes by which mutant *p53* exhibits its GOF are (1) through formation of mutant *p53* complexes with other proteins that modify their activities (e.g., *p63* and *p73*) and (2) by interaction of mutant *p53* with other transcription factors (e.g., *SREBP* and *Ets2*) that bring a potent transcriptional activation domain to promoters not normally regulated WT *p53* (Figure 1). These mechanisms are not necessarily mutually exclusive in the genesis of different cancers and may be context dependent (46, 47).

## Distinct Biological Activities of Different *p53* Mutants

In addition to exhibiting GOF phenotypes, mutant *p53* proteins exhibit intrinsic differences. Some are classified as structural mutants (e.g., *p53R172H*) as the mutation alters the structure of the DNA-binding domain while others are classified as DNA-binding mutants (e.g., *p53R245W* and *p53R270H*) because they alter an arginine that directly interacts with DNA. Other mutants show partial defects. For example, the *p53R172P* mutation, albeit rare, is able to activate the cell cycle arrest but not apoptotic programs of *p53* (48). *In vivo*, differences in tumor spectrum were observed between *p53^R172H^* and *p53^R270H^* mice (24, 25). In addition, in humanized mutant *p53* knockin models, *p53^R248Q/−^* and *p53^R248Q/Q^*, but not *p53^G245S/−^* and *p53^G245S/S^*, mice show an acceleration of tumor development and shorter survival as compared to *p53^−/−^* mice (49). Lastly, different human tumor types show different spectra of *p53* mutations. For example, based on cBioPortal, mutations at the codon 248 of *p53* are most prevalently observed in human pancreatic tumors, whereas in breast tumors, codons 275 and 175 are most frequently mutated, respectively (5, 6), further suggesting that different *p53* mutations impart unique activities to drive development of different tumor types.

## The Importance of Stroma in Tumor Suppression

The discussion has thus far focused on *p53* mutations within tumor cells and has ignored a possible role of surrounding tissue on tumor evolution. Tumors are complex tissues that consist of two components: a parenchyma and stroma. The parenchyma consists of tumor cells while the stroma consists of blood and lymphatic vessels, fibroblasts, and inflammatory and immune cells (50). The importance of stromal elements in cancer development has been supported by extensive clinical and experimental evidence (51–55). The injection of human breast tumors into nude mice and subsequent analyses of copy number variations indicated that stromal cells evolved additional changes not found in the original tumor (56). In another study of human breast cancer, gene expression differences in the stroma were a better predictor of response to chemotherapy (57). Mouse models have now clearly implicated the importance of stromal alterations in tumor development. Deletion of *PTEN* in stromal fibroblasts accelerated initiation, progression, and malignant transformation of *ErbB2/neu*-driven mammary epithelial tumors, implicating a tumor-suppressive role of *PTEN* in stroma (58). Global gene expression profiling of stroma lacking *PTEN* revealed changes in the expression of genes regulating extracellular matrix (ECM) deposition, wound healing, and chronic inflammation, which were validated by staining with various markers. Lujambio and colleagues selectively deleted *p53* in hepatic stellate cells, resulting in modifications to the tumor microenvironment (TME) and enhanced malignant transformation of epithelial cells (59). Mutations in *p53* have also been found in the stromal component of some primary breast tumors and in carcinoma-associated fibroblasts (CAFs) (51, 60–63). Additionally, MCF7 breast tumor cells formed more aggressive tumors with shorter latency after injection into *p53^−/−^* SCID mice as compared to injection into *p53*^+^*^/^*^+^ SCID hosts (64). Hill et al. (65) further showed that prostate tumor cells can promote the selection and expansion of *p53*-deficient stromal fibroblasts through paracrine mechanisms. Highly proliferative, *p53*-deficient stromal cells were subsequently found to promote epithelial tumor growth and progression despite retention of WT *p53*. These data clearly show that changes in stroma occur and that they directly impact tumor development.

## Mutant *p53* Addiction

In addition to the observations that mutant *p53* proteins exhibit GOF activities, a growing body of evidence suggests that tumor cells may be addicted to mutant *p53* expression. Experiments using siRNA knockdown of mutant *p53* in cancer cell lines showed a higher apoptotic response to drug treatment in cells with knockdown of mutant *p53* (47, 66). Additional mutant *p53* depletion experiments show decreases in cell growth rate, viability, replication, and clonogenicity. Constitutive inhibition of mutant *p53* reduced tumor growth in nude mice and showed reduced stromal invasion and angiogenesis (67). In addition, Prives and colleagues showed that mutant *p53* depletion in breast cancer cells (MDA-MB-231 cells with *p53*^*R280K*^ and MDA-MB-468 with *p53*^*R273H*^) in 3D culture leads to phenotypic reversion to more normal, differentiated structures with hollow lumens (43). More recently, using a conditional mutant *p53* mouse model expressing a *p53*^*R248Q*^ hotspot mutation, Moll and colleagues showed that mutant *p53* ablation restrained growth of allotransplanted and autochthonous tumors and extended animal survival, indicating that these tumors depend on sustained mutant *p53* expression (68). In summary, these data suggest that tumor cells with mutant *p53* may be addicted to the GOF activities of mutant *p53*. Tumor regression and dependence on mutant *p53* is likely context dependent and the extent to which elimination of mutant *p53*, genetically or through pharmacologic inhibition of downstream mediators, as a viable therapy remains to be seen (69).

## Mouse Models for Sporadic *p53* Mutations in Cancer

Knock-in mice with germline mutations in *p53* that mimic those found in LFS have more aggressive, metastatic tumors as compared to mice lacking *p53* and provided convincing evidence for *p53* GOF activities (24, 25). Yet germline mutations in *p53* are rare in humans, and the vast majority of human cancers evolve from somatic alterations of *p53*. Consequently, current animal models at our disposal to study the role of *p53* missense mutations in the genesis of somatic tumors are inadequate. Some involve expression of mutant *p53* in breast epithelial cells using mouse mammary tumor virus (MMTV) or whey acidic protein (WAP) promoters which are hormonally regulated and therefore do not simulate expression of mutant *p53* from the endogenous locus (70, 71). Currently, conditional mutant *p53* alleles are only active following cre-mediated removal of a DNA “STOP” cassette flanked by LoxP sites (LSL = Lox–STOP–Lox) (25). The STOP sequence maintains the downstream gene in an unexpressed or null state in all animal cells until the STOP cassette is selectively removed and mutant *p53* is expressed. Importantly, tumors in this model initiate from cells heterozygous for *p53* since animal conception. Moreover, tumors under these conditions initiate and progress within a tumor microenvironment (TME) replete with *p53*-heterozygous stromal elements. CAFs, macrophages, T cells, neutrophils, endothelial cells, and so on remain heterozygous for *p53* following conception (due to the presence of the STOP cassette) with undefined effects on tumor biology. Stroma is a requisite component of tumor initiation and growth, and as mentioned earlier, prostate tumor epithelium selects for *p53*-null stromal fibroblasts in *p53*^±^ mice, yielding a highly proliferative stroma that contributes to tumor progression. Given the powerful roles of *p53* in cellular plasticity and embryonic stem cell differentiation, tumors that develop from and under conditions of *p53* heterozygosity may differ in unappreciated ways from human tumors that initiate from WT *p53* cells. A model that more closely mimics the events in sporadic tumorigenesis is sorely needed.

## Future Directions

Mouse models of mutant *p53* carry the potential to inform us of essential mechanisms of cancer initiation and metastasis translatable to therapeutics in humans. However, many genetic mouse models used to study mutant *p53 in vivo* incorporate germline mutant *p53* alleles that may alter normal and cancer biology in ways that compromise its relevance to human cancer. Innovation in genetic mouse models of mutant *p53* is mandatory to more closely model human biology and to serve as translational biologic platforms to better evaluate and develop novel therapeutic agents in human cancers. Moreover, given the importance of the TME in cancer development and metastasis, mouse models that preserve the complex regulatory and tumor–stromal interactions are mandatory to the development of effective, translational biologic platforms to target the TME toward therapeutic ends.

## Author Contributions

MK, YZ, and GL wrote and edited the manuscript. MK and YZ generated the figure.

## Conflict of Interest Statement

The authors declare that the research was conducted in the absence of any commercial or financial relationships that could be construed as a potential conflict of interest.
